# Supplementary figures and images for: Genome-Wide Association and Genomic Prediction of Growth Traits in the European Flat Oyster (Ostrea edulis)
Source: Front Genet. 2022 Jul 15;13:926638. doi: 10.3389/fgene.2022.926638 (PMC9380691; doi:10.3389/fgene.2022.926638)

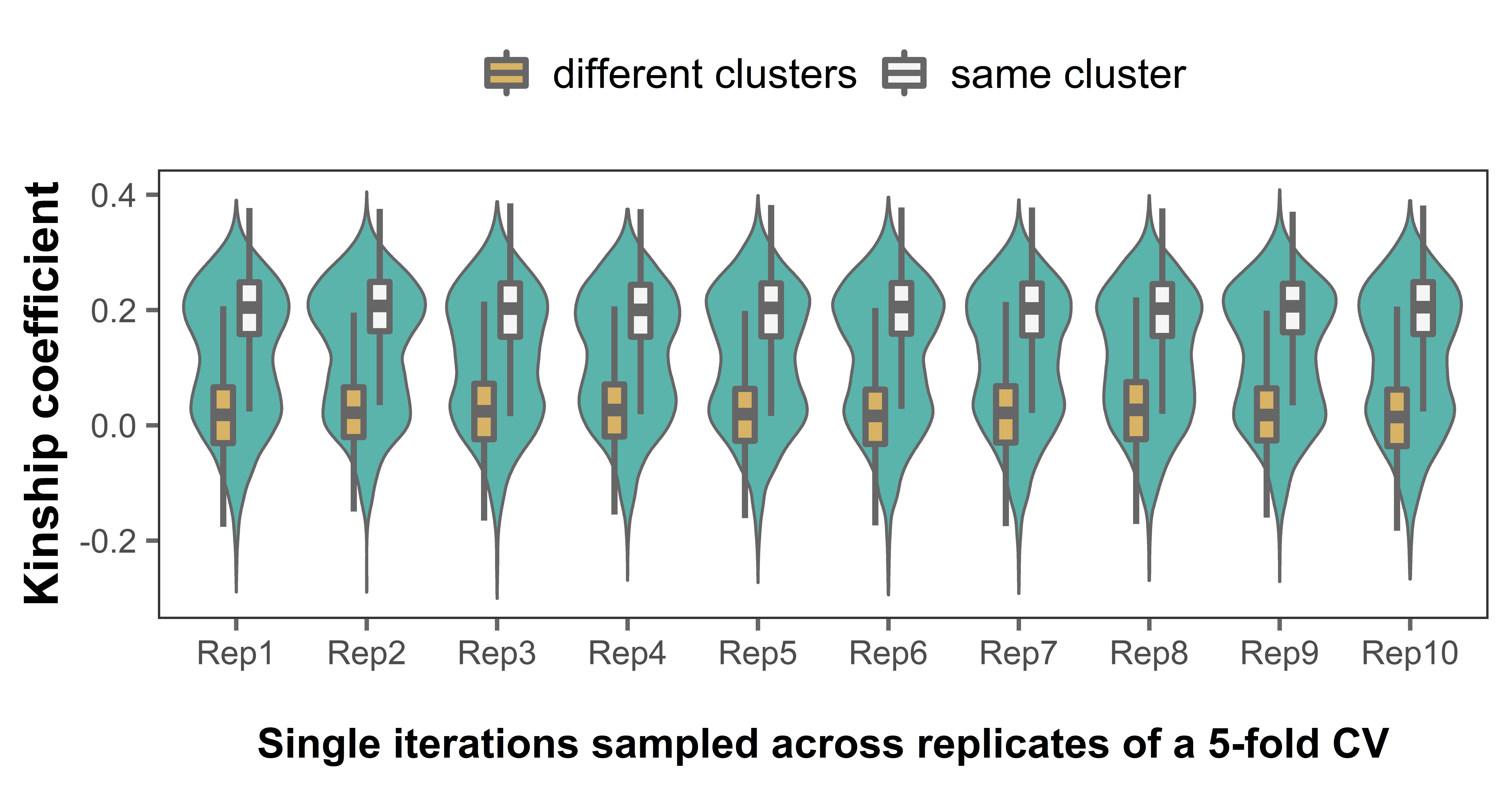

Supplement: Supplementary file 2 [file Image3.JPEG]

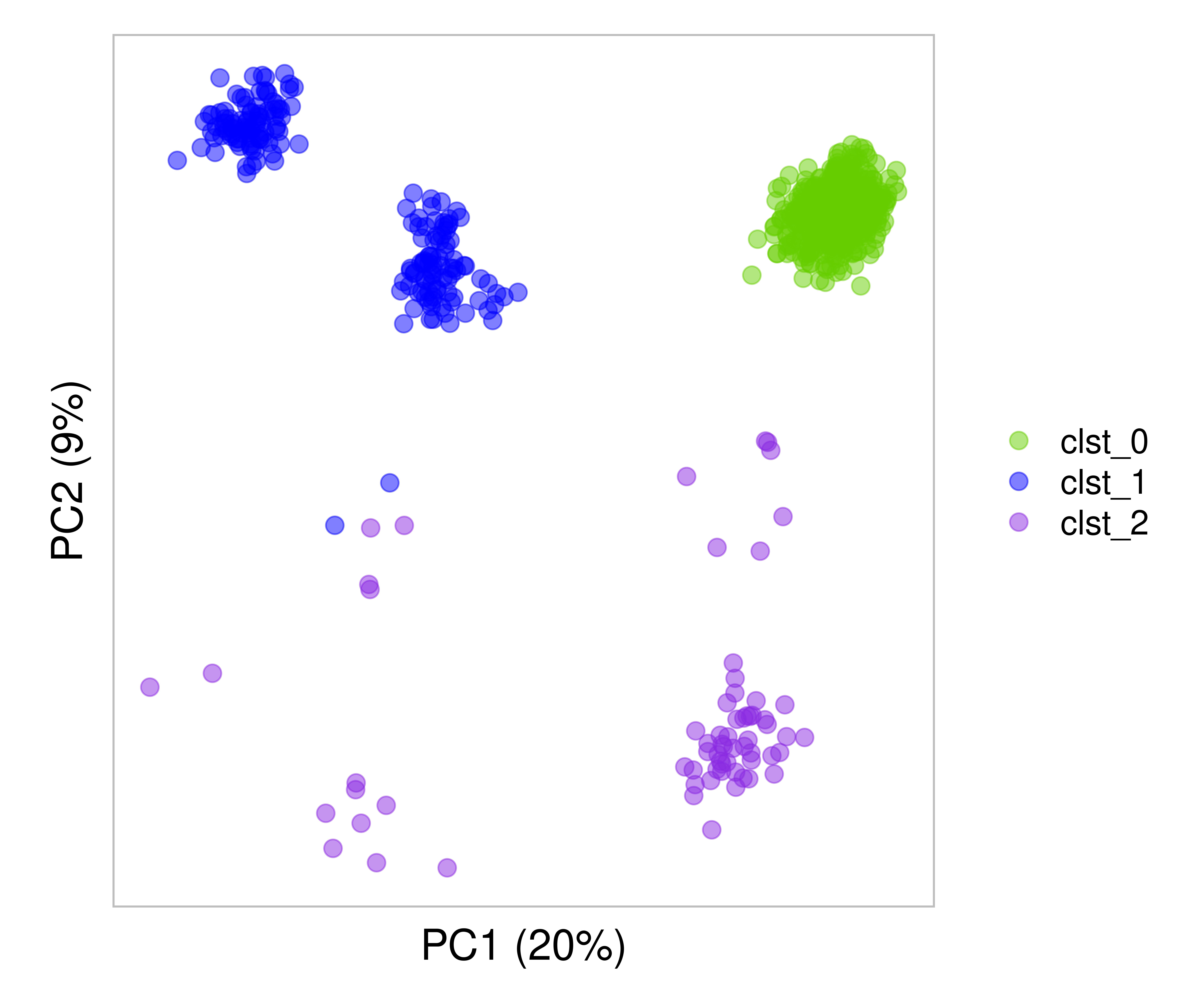

Supplement: Supplementary file 4 [file Image1.jpeg]
